# Intelligent Reflecting Surfaces Assisted UAV Communications for Massive Networks: Current Trends, Challenges, and Research Directions

**DOI:** 10.3390/s22145278

**Published:** 2022-07-14

**Authors:** Syed Agha Hassnain Mohsan, Muhammad Asghar Khan, Mohammed H. Alsharif, Peerapong Uthansakul, Ahmed A. A. Solyman

**Affiliations:** 1 Ocean College, Zhejiang University, Zheda Road 1, Zhoushan 316021, China; hassnainagha@zju.edu.cn; 2 Department of Electrical Engineering, Hamdard Institute of Engineering & Technology, Islamabad 44000, Pakistan; m.asghar@hamdard.edu.pk; 3 Department of Electrical Engineering, College of Electronics and Information Engineering, Sejong University, Seoul 05006, Korea; 4 School of Telecommunication Engineering, Suranaree University of Technology, Nakhon Ratchasima 30000, Thailand; 5 Department of Electrical and Electronics Engineering, Faculty of Engineering and Architecture, Nişantaşı University, Istanbul 34398, Turkey; ahmed.solyman@nisantasi.edu.tr

**Keywords:** intelligent reflecting surface (IRS), passive reflections, radio frequency, unmanned aerial vehicle, IRS-assisted UAV

## Abstract

An intelligent reflecting surface (IRS) can intelligently configure wavefronts such as amplitude, frequency, phase, and even polarization through passive reflections and without requiring any radio frequency (RF) chains. It is predicted to be a revolutionizing technology with the capability to alter wireless communication to enhance both spectrum and energy efficiencies with low expenditure and low energy consumption. Similarly, unmanned aerial vehicle (UAV) communication has attained a significant interest by research fraternity due to high mobility, flexible deployment, and easy integration with other technologies. However, UAV communication can face obstructions and eavesdropping in real-time scenarios. Recently, it is envisaged that IRS and UAV can combine together to achieve unparalleled opportunities in difficult environments. Both technologies can achieve enhanced performance by proactively altering the wireless propagation through maneuver control and smart signal reflections in three-dimensional space. This study briefly discusses IRS-assisted UAV communications. We survey the existing literature on this emerging research topic for both ground and airborne scenarios. We highlight several emerging technologies and application scenarios for future wireless networks. This study goes one step further to elaborate research opportunities to design and optimize wireless systems with low energy footprint and at low cost. Finally, we shed some light on open challenges and future research directions for IRS-assisted UAV communication.

## 1. Introduction

In the last decade, we have seen innovative breakthrough from both academia and industrial sectors to launch fifth-generation (5G) and envision the future roadmap of sixth-generation (6G) technology. 6G is envisioned to support high coverage extension, massive connectivity, higher data rates, lower latency, and higher reliability compared to 5G. Several research efforts have been made using diversity techniques, modulation schemes, and channel coding to secure ultra-reliable wireless communication. However, these strategies are unable to alter the wireless propagation, which arises the need for novel mechanisms to achieve smart, intelligent, reconfigurable and controllable wireless propagation. It is possible through UAV communication, IRS communication or IRS-assisted UAV communication. 

Due to high flexibility, mobility, and cost-effectiveness of UAVs, UAVs play an integral role in existing wireless networks. UAVs are currently being used in disaster-stricken areas, such as floods and earthquake monitoring where terrestrial networks are damaged [1]. UAVs have been used to monitor traffic and supply first aid medical supplies. In wireless communication, UAVs trajectories can be intelligently adjusted to achieve reliable communication. UAVs support enhanced channel conditions by offering line-of-sight (LoS) links. Communication link quality can be further improved by intelligently controlling hovering or trajectories to reduce the distance range for LoS path. Furthermore, UAV’s high mobility also enables improved performance for air to ground networks. Several studies have been conducted to optimize UAV’s trajectory to satisfy quality of service (QoS) requirements [2]. To achieve high capacity and better performance, UAVs can be integrated with emerging technologies such as wireless power transfer (WPT), non-orthogonal multiple access (NOMA), visible light communication (VLC), machine learning (ML), millimeter wave (mmWave), etc. Several works have been reported to support stable performance of UAVs targeting throughput, capacity, achievable data rate, security, coverage, and reliability. Several studies have focused on UAV communication [3,4,5]. Several projects and other initiatives are being introduced to explore breakthrough novelties assisted by UAV communication. However, there are multiple research areas which require further contributions to implement UAV communication in real-time. For example, UAV size, payload, mission time, and battery endurance [6] are subject to performance degradation and make it difficult to incorporate UAV with other emerging technologies such as intelligent reflecting surface (IRS) and multiple-input and multiple-output (MIMO) to achieve flexible beamforming and reliable communication along with reduced path loss. The most crucial concerns are stringent size, weight, and power (SWAP) constraints. Additionally, LoS link blockage due to high rise buildings [7] and high power consumption to ensure airborne condition and high mobility also pose critical limits. Similarly, security and privacy issues of legitimate network entities can suffer from potential eavesdroppers. These problems can be alleviated by using promising IRS.

**Figure 1:**
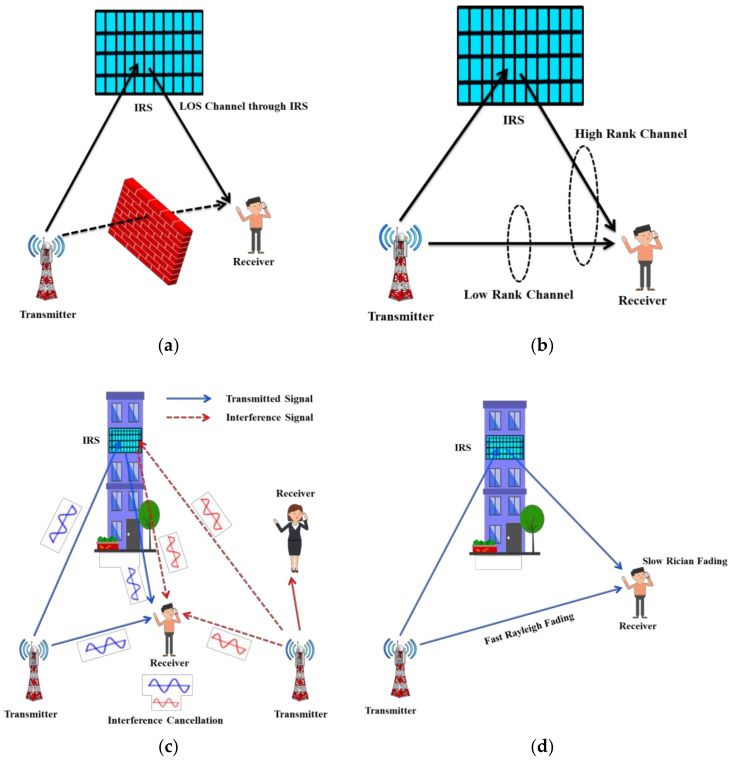
Main functionalities of IRS to reconfigure wireless propagation. (**a**) IRS for coverage extension; (**b**) IRS to enhance channel rank condition; (**c**) IRS to refine channel statistics; (**d**) IRS to suppress interference.

IRS, a novel paradigm, can smartly configure the wireless propagation to ensure smart wireless communication [8,9]. It is a large planar surface with a large number of passive reflection elements to change amplitude, frequency or phase of impinging signals. It enables a novel mechanism to configure wireless channel through a quantum leap gain for capacity, reliability, sustainability, coverage, and secrecy performance of wireless communication. IRS has the potential to enhance energy efficiency and physical layer security as well [10,11]. It is more energy efficient as the phase, absorption, reflection, or refraction of IRS passive reflecting elements can control the incident signals without any need of RF chains. As compared to active relying system or backscatter communication, IRS can be easily installed at buildings, vehicles, or indoor walls with minimal expenditure and efforts. In addition to above benefits, IRS supports the advantage of integration and compatibility with current radio techniques. Some features of IRS to configure wireless communication are illustrated in Figure 1. Figure 1a shows IRS for coverage extension, while Figure 1b represents IRS to enhance channel rank condition. Similarly, IRS for channel statistics refining and interference suppression are illustrated in Figure 1c,d. 

**Figure 2:**
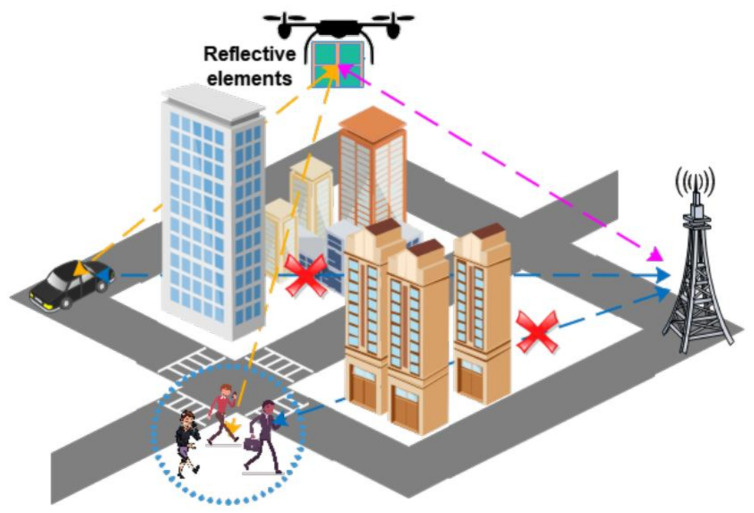
IRS-assisted UAV communication [23].

IRS offers high coverage, flexible deployment and extended range of signal reflection when it is integrated on a mobile UAV rather than a fixed wall or building. Along prior potential capabilities of IRS, half duplex and full duplex communication can also be achieved over wide bandwidth and frequency. Due to its uniform spatial configuration, installed on high altitude buildings, IRS can provide shorter LoS paths with UAVs and blocked users through phase shift. Furthermore, IRS-assisted UAV can substantially reduce the channel complexity and interference for wireless communication as well as access delay and propulsion energy consumption for UAVs as illustrated in Figure 2. Additionally, IRS can enhance channel quality in urban scenarios along with mitigating adverse eavesdropping [12,13]. IRS-assisted UAV can significantly reduce power consumption by joint optimization of UAV’s trajectory and resource allocation [14]. 

Several works have been reported on IRS-assisted UAV communication [15,16,17,18,19,20,21,22]. A recent study [23] highlighted various applications, challenges and prospects for IRS-assisted UAV communication for cellular networks considering giant-site access, spectrum sharing, extended coverage, and physical layer security (PLS). Another study carried out performance analysis of IRS-assisted UAV considering both aerial and terrene IRS [24]. Most of the existing literature has focused on a fixed IRS limited to nearby users. While some works are reported on multi-IRSs to bypass any obstruction causing high path loss [25,26]. To overcome these challenges, an alternative approach is to utilize IRS-assisted UAVs for wireless communication. Table 1 outlines existing literature on IRS-assisted UAV communication.

### 1.1. Advantages of IRS-Assisted UAV Communication

**Figure 3:**
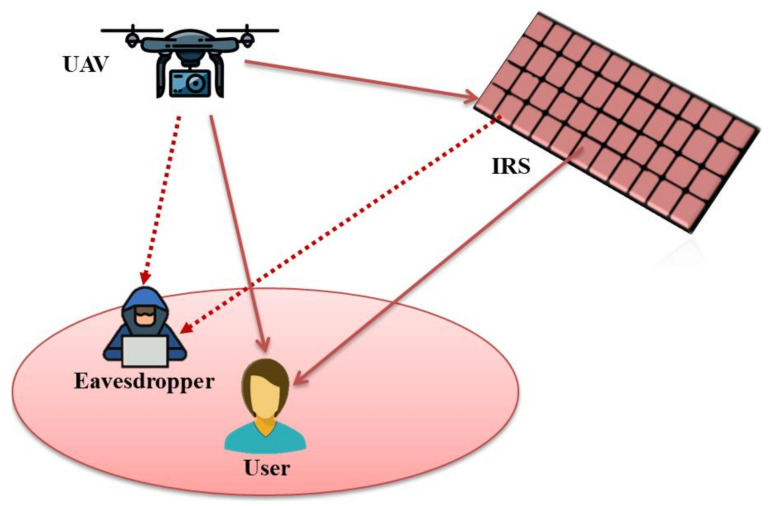
IRS-assisted UAV communication in the presence of eavesdropper.

Some of the key advantages of IRS-assisted UAV communication are as follows:

✓ In case of absence of LoS links due to any blockage between UAV and ground users, virtual LoS links can be established through IRS. Therefore, communication quality can be enhanced, which further extends the wireless network coverage;
✓ When IRS is installed on a moving UAV, a new degree of freedom can be obtained for IRS as UAV’s mobility and IRS’s position can be dynamically configured;
✓ When IRS is installed on a moving UAV, a direct LoS link can be designed between transmitter and receiver. By installing IRS on a UAV, full-angle reflection can be obtained which further extends network coverage;
✓ Both IRS and UAV can operate in mmWave and terahertz (THz) band, empowering high data rate and ultra-bandwidth. As mmWave and THz links are prone to blockage and high path loss, IRS’s reconfigurability and UAV’s mobility can effectively compensate these issues;
✓ IRS-assisted UAV communication can significantly improve the achievable data rate signal-to-noise-ratio (SNR). A recent study considers ground-based IRS to improve ground-air UAV communication [27];
✓ IRS-assisted UAV can effectively improve communication quality and data rate as IRS can be easily integrated with promising technologies, such as free-space optics (FSO) and non-orthogonal multiple access (NOMA);
✓ The UAV communication links are always observable to potential eavesdroppers. Thus, reliability of communication links can be hampered by channel interference caused by these eavesdroppers. To compensate this issue, IRS can be involved to reduce external interference. In [28], the authors address IRS-assisted UAVs for two different applications in cellular networks: (i) to reduce the secrecy to avoid eavesdropping, and (ii) to maximize the average users’ achievable rate. Figure 3 shows IRS-assisted UAV communication in the presence of an eavesdropper. In this scenario, the potential eavesdropper can steal information of user and can cause other security concerns. Through IRS passive beamforming, it is possible to strengthen the reflected signals for the legitimate user as well as reduce the received SNR at the eavesdropper. Hence, we can improve the reliability and PLS for UAV communication. Similarly, airborne IRS can also offer a significant performance improvement for the intended user by optimizing UAV’s location and trajectory;
✓ In UAV communication, energy consumption is a crucial factor due to limited payload and batteries. Some recent studies have addressed IRS-assisted UAV communication to tackle the energy consumption challenges. In [29,30], authors have focused on join optimization for UAV trajectory, power allocation and IRS phase shift in order to decrease the average energy consumption. Another study [31] introduces extended probabilistic LOS model to unlock the potentials of IRS-assisted UAV communication. In [32], authors consider a directional antenna on a cooperative UAV pointing towards IRS to reduce the energy consumption. We have summarized some existing studies on IRS-assisted UAV communications in Table 2.

### 1.2. Scope and Contributions

This review is devoted to IRS-assisted UAV communication and will help readers to understand ongoing research and development activities on this promising integration of these promising technologies. Our goal is to support readers to get an overview of IRS, structure, working principle, and UAV etc.

- We provide comprehensive review of advantages of this coexistence, and the use cases where this coexistence of UAVs and IRSs can be advantageous;
- We carry our extensive analysis to empower the use of this integration in several applications scenarios;
- In the end, we discuss several potential challenges and future research directions to further hones the research work dedicated to this promising integration of IRSs and UAVs.

### 1.3. Organization of the Paper

We have structured this study as follows: Section 2 provides an overview of the research contributions on UAVs and IRSs. Section 3 presents the emerging technologies or use cases which can benefit from this integration. Section 4 briefly discusses the application scenarios of IRS-assisted UAV communications. We examine potential challenges and future research directions in Section 5. Finally, this paper is concluded in Section 6. 

## 2. An Overview of IRS and UAV Technologies

This section sheds some lights on promising IRS and UAV technologies. For IRS, we have discussed basic architecture and working principle. We have focused on common architecture which is presented is different studies on IRS. For, UAV, we have addressed on existing studies and products. 

### 2.1. IRS Technology

#### 2.1.1. Basic Architecture of IRS 

**Figure 4:**
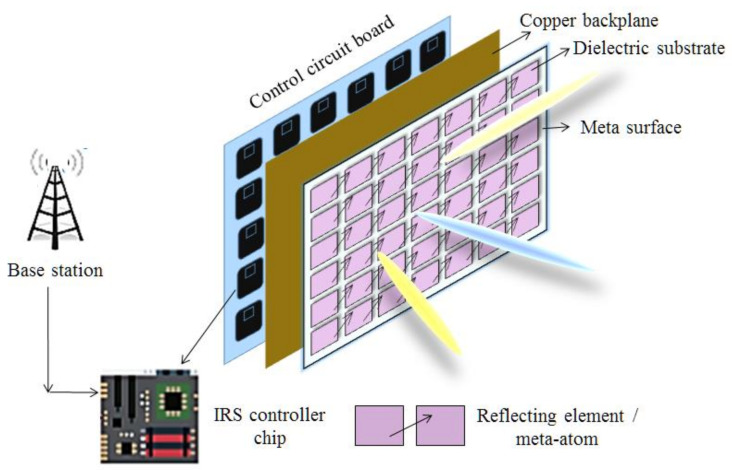
Basic hardware architecture of IRS.

The architecture of IRS is a viable phenomenon. The existing studies on IRS suggest different designs composed of different number of layers. Generally, IRS architecture is based on liquid crystals, varactor-tuned resonators, microelectromechanical systems (MEMS), doped semiconductor, and electromechanical switches. Despite the fact that different designs are available in published studies, most of these studies focused on architecture of three layers: (i) a metasurface layer, comprised of passive conductor; (ii) a control-layer, used to alter the amplitude or phase of meta-atom elements; and (iii) a gateway layer which can communicate between control layer and a BS. The basic operational function of each meta-atom is to adjust EM characteristics as a sub-wavelength scatterer. This feature helps to entirely change the incident patterns on IRS into required EM response [37]. It supports IRS to configure the wavefronts in order to achieve beam steering, absorption, filtering, localization, collimation, sensing, polarization and filtering. Two basic methods to achieve focusing and steering are conventional antenna arrays and metasurfaces [38]. Figure 4 presents the basic hardware architecture of IRS. In Table 3, we have summarized existing studied on reconfigurable metasurfaces. 

#### 2.1.2. Working Principle 

**Figure 5:**
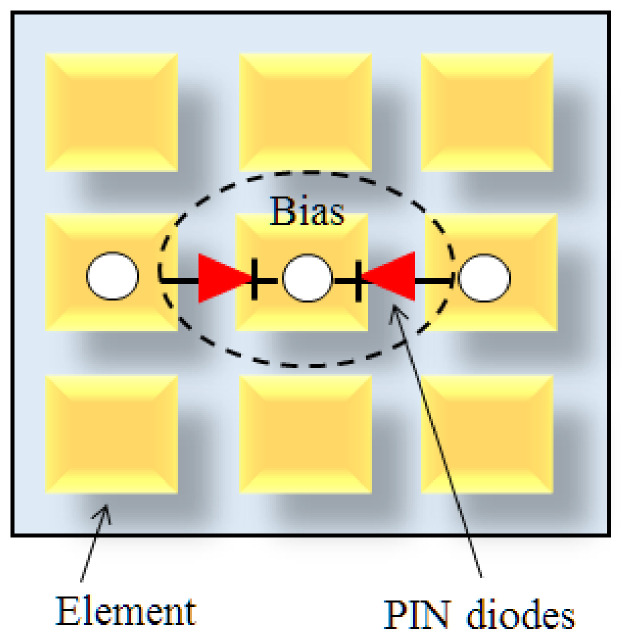
EM reflection via PIN diodes.

Generally, IRS is a two-dimensional array based on massive number of passive reflecting elements. IRS operates on the basic principle of altering signals through reflection and altering their phase shifts. In this way, IRS can attain cost-effective sustainable performance [45]. In some recent studies, authors have focused on intelligent reflecting surfaces, large intelligent surfaces antennas, compared with relaying systems and roles in future 6G Internet-of-Things (IoT) networks [46]. In [47], R. Alghamdi et al. investigated passive reflecting elements or metasurfaces in large intelligent surface (LIS). These surfaces are composed of complementary metal-oxide-semiconductor (CMOS) switches or microelectro-mechanical systems (MEMS) with the capability to smartly alter phase shifts of incident waves. Some other switching technologies, e.g., positive intrinsic- negative (PIN) diodes and liquid crystal, are also available. In terms of PIN diode, PIN on off condition is achieved through an external bias, which can create two distinct levels as shown in Figure 5. In this context, maximum portion of energy is reflected as PIN diode is on, while maximum energy is absorbed as PIN diode is off. 

The electromagnetic characteristics of metasurfaces are closely related to meta-atom patterns. Therefore, some structure of meta-atoms can partially reflect while others can completely reflect the incident EM signals. These metasurfaces comprised of passive reflecting components are dynamic in nature; thus, these surfaces can select required EM response or an external bias can adjust their conditions. The scattering elements of these surfaces can act as input or output antennas. Thus, when an incident EM signal enters from the input antenna, transmission occurs on the basis of switch status, and this EM signals exits from the output antenna with a desired reflection. Table 4 illustrated several projects from literature.

**Figure 6:**
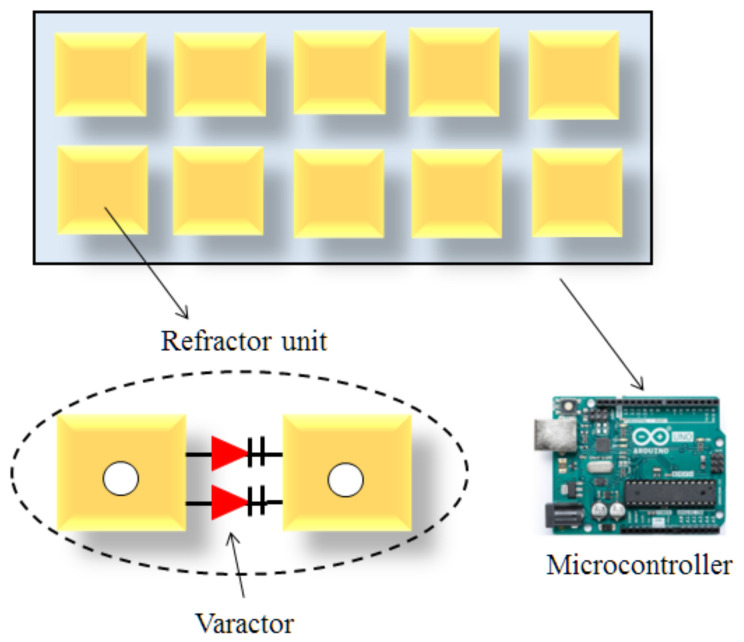
Controlling EM reflection via varactor-tuned resonators.

Apart from above discussed methods, varactor-tuned resonators are also suggested to configure the incident EM waves as illustrated in Figure 6. In this case, bias voltage is provided to the varactor with the objective to achieve phase shift. This attained phase shift can be further controlled by liquid crystals. Effective dielectric constant can be achieved by changing the DC voltage on liquid crystals. Through this technique, the phase shift of impinging signals can be controlled at different points of the metasurface. 

### 2.2. UAV Technology 

UAVs refer to controlled aerial vehicles which perform several missions without human assistance. They can be remotely operated through different electronic gadgets such as microprocessors and sensors. UAVs can perform operation autonomously in such areas where human intervention is dangerous. The architecture of UAV usually utilizes communication links to establish connectivity with satellite or ground control system (GCS) such as laptop or smart phone. A human operator is needed to operate and control UAV remotely. In literature, several works have been presented to address different features of UAVs. Several recent studies have focused on various aspects of UAVs such as adaptive UAV deployment [54], drone collaboration [55], and mobile charging features [51]. Some researchers have addressed the use of wireless powered receivers in order to decrease the weight of UAVs. Several wireless charging techniques have been suggested to increase the UAV flight time. Shin et al. [56] addressed charge scheduling for multi-drones’ networks based on deep learning algorithms. In Table 5, we have summarized different surveys and reviews reported on multiple aspects of UAVs.

**Figure 7:**
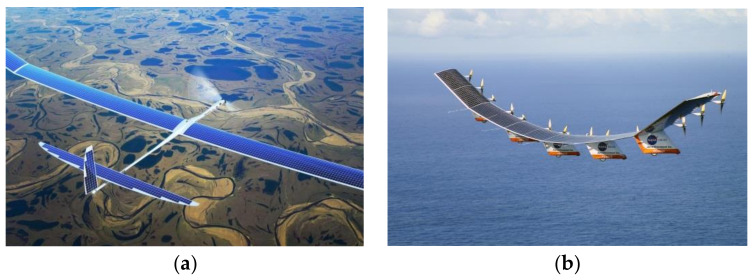
Solar-powered drones (**a**) Google’s project, (**b**) Facebook’s project [65].

Solar-powered UAVs have gained significant attention in both academia and industrial sectors. Several research and industrial activities have been initiated on solar-powered UAVs. Figure 7 shows solar-powered drones projects by technology giants e.g., Google and Facebook. Google started using these UAVs to provide internet access in remote areas. At present, such UAVs are being used for forest fire fighting, internet coverage, high altitude communication, border monitoring, and power-line inspection. 

## 3. Emerging Technologies

IRS-assisted UAVs offer a highly configurable approach to extend the two-dimensional network model to the three-dimensional model, satisfying the requisites of future wireless networks. It can provide reduced energy consumption, secure transmission, high reliability, extended coverage, enhanced QoS and tuned channel gains. The future networks with aforementioned benefits will provide a continual stream of breakthrough innovations to sit the needs of cutting-edge applications and services. There are multiple networking and communication use cases where the coexistence of UAVs and IRSs can be advantageous. Here, we disclose the impacts that this framework can have on machine learning (ML), mmWave, THz, WPT, FSO, VLC, NOMA, and IoT. 

### 3.1. Machine Learning 

The RIS-assisted UAV technology is a promising technology to support various applications. Novel breakthroughs can be achieved through artificial intelligence (AI) and machine learning (ML). These transformative technologies can empower IRS-assisted UAV communication in terms of QoS, reliability, security, and network performance. The IRS-assisted UAV can provide potential features like intelligent and controlled decision-making, data extraction and forecasting and optimal optimization. The learning can be implemented using supervised, unsupervised or reinforcement learning. ML tools can be utilized to improve IRS channel estimation, embedded optimization, UAV tracking performance, spectral efficiency, and managing various tradeoffs. The ML tools can be used to control UAV’s location and IRS’s phase shift to achieve desired communication. Accurate tracking and reliable channel conditions can be obtained through ML algorithms. Convolutional neural networks (CNNs) and deep neural networks (DNNs) are used to reduce the complexity and computational time of these algorithms. Next, we have discussed research contributions on IRS-assisted UAV communication using deep learning. 

#### Deep Learning 

Recently, deep learning (DL) algorithms have gained significant attention in wireless communication. These algorithms offer a decisive benefit over conventional methods. It is gained through a neural network framework of several layers with the capability to discover latent structure with labeled, unlabeled, and unstructured data by processing it in an unsupervised, supervised, reinforcement, or hybrid manner. DL-assisted solutions have been deployed for network routing, proactive caching, resource optimization, and dynamic reconfiguration of antenna tilts to optimize coverage. DL-driven approaches show a strong feature to replace traditional modulation and coding schemes, enabling smart, intelligent, and pro-active adaptation to the environment. Several DL-enabled solutions have been proposed to find the optimal reconfiguration of metasurfaces. Similarly, deep reinforcement learning (DRL) algorithms are proposed as a viable solution instant decision-making and embedded optimization in wireless communications. The DRL algorithms are utilized for IRS-empowered wireless networks [66] and UAV-aided networks [67]. In [67], the authors introduced a DRL approach to optimize the IRS shift in order to enhance the SNR. Furthermore, authors in [68] consider DRL algorithm to optimize phase shift of IRS and transmit beamforming vector for maximizing the total sum-rate. In [69], authors proposed a DRL algorithm to control the IRS phase shift and UAV’s altitude to reduce the sum age-of-information. However, these works consider static environment, flat fading channel setting, perfect CSI or idealistic conditions. These conditions are infeasible and unrealistic for practical use cases. Additionally, the delay in both centralized learning and processing time to optimize algorithm is also longer for practical applications. In [7], researchers introduced proposed a deep deterministic policy gradient (DDPG) algorithm to investigate DRL applications to IRS-UAV NOMA downlink system. The proposed technique is utilized to optimize the IRS phase shift, horizontal position of UAV and power allocation of BS. The presented results show better performance in terms of robustness and sum rate. An efficient DRL approach is proposed in [70] to jointly optimize UAV’s 3D trajectory and IRS phase shift to improve the communication quality. In [71], authors proposed IRS-assisted transmission strategy to address the link performance and coverage of aerial terrestrial communication. Particularly, authors designed an adaptive IRS-assisted transmission protocol based on data transmission, transmission strategy and channel estimation. Authors formulated an optimization problem through multi-task learning to enhance the entire system’s capacity. The proposed strategy reduced the transmit power and enhances the system throughput.

### 3.2. Mm-Wave and THz

Future 6G technology is envisaged to utilize high frequency bands of tens of GHz bandwidths, such as mmWave and THz. It will necessitate technologies and network architectures different from existing cellular networks. Thz and mmWave communication experience severe path loss peaks due to harsh atmospheric conditions, such as molecular absorption and attenuation [72]. Furthermore, considering these technologies of higher bandwidths will support the blockage rate of transmission, severely affecting the availability and reliability of wireless network. In case of short-range LoS settings, THz communication has shown good performance. To overcome these challenges, IRS-assisted UAV technology is a viable approach. In this scenario, a UAV can be used for short range communication with THz band transmitter, while IRS elements are used to configure the channel conditions to achieve optimum signal links for the outbound links. Severe propagation attenuation can be alleviated by deploying IRS in THz communications. Several innovative efforts show that IRS can be used in THz communication to maximize sum-rate performance [73] and maintain reliability [74]. However, these techniques are usually implemented for terrene THz communication, and we cannot directly implement for aerial THz communication. Furthermore, the path loss in THz is closely associated to link distance, which greatly relies on UAV trajectory. Thus, it is imperative to collaboratively optimize sub-band allocation, IRS phase shift and UAV’s trajectory to improve the ITS-assisted UAV supported THz communication. In a recent letter [75], authors have addressed UAV-supported THz communication where IRS is also used to help the transmission. Authors formulated optimization problem for power control, THz sub-band allocation, IRS’s phase shift and UAV’s trajectory. 

Due to the blockage-prone feature of mmWave signals, dynamic IRSs are more suitable than static IRSs to improve mmWave communication. A UAV-carried IRS can be used to constantly adjust the position of IRS in order to maintain persistent LoS paths between transmitter and receiver. Being installed onto a UAV, an IRS can significantly support reliable of mmWave communication by smartly optimizing its location. In [22], authors proposed a novel framework to effectively deploy UAV-IRS to support mmWave downlink transmission in a dynamic scenario. The proposed mechanism shows better performance as compared to static IRS. The authors in [76] address the secure communication in a UAV-IRS aided mmWave network in the presence of an eavesdropper. This study shows enhanced secrecy rate than benchmark schemes. While in [77], authors proposed a resource allocation algorithm for mmWave communication using multi-UAVs-borne IRSs.

### 3.3. Wireless Information and Power Transfer

WPT has emerged as a promising technology to prolong the battery life of IoT tags or UAVs. These technologies are prone to limited battery endurance. To overcome the issue of limited battery or high power consumption, several techniques have been introduced including waveform design, energy transmission, scheduling, energy beamforming, and wireless energy harvesting. Another alternative approach is to deploy IRS close to or carried by UAVs to support efficient energy charging. By incorporating IRS, the received power can be significantly enhanced. Moreover, the advancements in WET can enhance the efficiency ensuring flexibility in beamforming which further enhances the performance of SWIPT systems [78]. SWIPT is emerging techniques to avoid frequent battery replenish or recharging in difficult scenarios such as in underwater applications or in concrete structures for embedded monitoring sensors, flying drones, and in implantable medical sensors for human bodies. IRS’s passive beamforming can support WPT and SWIPT systems as it offers additional LoS paths to improve the harvested power. Several researchers have demonstrated communication environment achieved by IRS to support existing wireless communication techniques in terms of efficiency, resource allocation, max-min fairness, sum-rates maximization, weighted sum-power etc., to benefit WPT and SWIPT. In [79], authors reported UAV-IRS assisted SWIPT for IoT networks. The presented results show that using reliable and flexible UAV-IRS, the minimum achievable rate can be effectively enhanced. In [80], a novel IRS-UAV empowered SWIPT framework is proposed. The proposed framework utilizes IRS to design the wireless channel aiming to enhance the WET efficiency and extend coverage area of SWIPT network. In this study, authors formulated optimization problem considering IRS’s reflection coefficient, power splitting, UAV power allocation and trajectory while taking account of energy harvesting and NOMA user. Another recent study [81] addresses IRS-aided SWIPT system. In this system, multi-IRSs are mounted on ground buildings and UAVs are used to improve the SWIPT. The optimization problem is designed considering UAVs trajectories, IRS phase shifts, transmit beamforming, and power splitting to effectively increase the average achievable rate. 

### 3.4. Visible Light Communication (VLC)

VLC is a promising candidate to meet the requirements of B5G or 6G technology as it supports low implementation cost, low-cost components, high data rate, simple design, easy deployment, and license free spectrum. However, it is prone to various impairments such as limited coverage, pointing errors, misalignment, and strict LoS requirement. To mitigate these shortcomings, IRS-assisted UAV communication is proposed. The meta-lens or crystal liquid-based IRS can be utilized to control the incident optical beam. These kinds of IRSs can effectively control the optical beam through dynamic artificial muscles, controlling IRS thickness or refractive index [23]. The IRS passive reflecting elements can smartly control the incident optical beam by adapting the required channel configuration. Similarly, fast and accurate alignment can be achieved through UAV’s mobility. 

### 3.5. Non-Orthogonal Multiple Access (NOMA)

UAVs support service to a large number of nodes with stringent requirements in UAV communication. Advance multiple techniques are used to satisfy these requirements. Specifically, NOMA is a promising candidate due to enhanced spectral efficiency, massive connectivity, and user fairness guarantee. NOMA enables multiple users to compromise over communication resources such as frequency, time, space, and spreading code by invoking successive interference cancellation (SIC) and superposition coding (SC) techniques. In future, efficient algorithms must be developed to fully reap the potentials of NOMA in IRS-assisted UAV which is commonly conceived as an innovative strategy due to following reasons:

- Firstly, NOMA ensures high flexibility and efficient resource allocation for IRS-assisted UAV communication as compared to traditional orthogonal multiple access (OMA). Thus, it can provide enhanced spectral efficiency as well as satisfying diversified communication demands of network users;
- Secondly, the traditional NOMA performs SIC decoding by considering strong and weak users on the basis of channel conditions. These channel conditions can be configured by controlling UAV’s mobility and IRS phase shift to empower a smart NOMA operation. This smart integration of technologies has been addressed in some recent studies. For instance, the authors [82] discuss NOMA to serve multiple users to attain a flexible and high coverage performance of a cellular network. This study analyzes optimal transmit power allocation between UAVs and two NOMA users to ensure flexible and ubiquitous NOMA transmission. In [83], the authors highlight the performance of IRS-assisted UAV NOMA system to serve multiple ground users. The authors discuss the potentials of integrating these three technologies to effectively improve the system performance.

### 3.6. Mobile Edge Computing (MEC)

**Figure 8:**
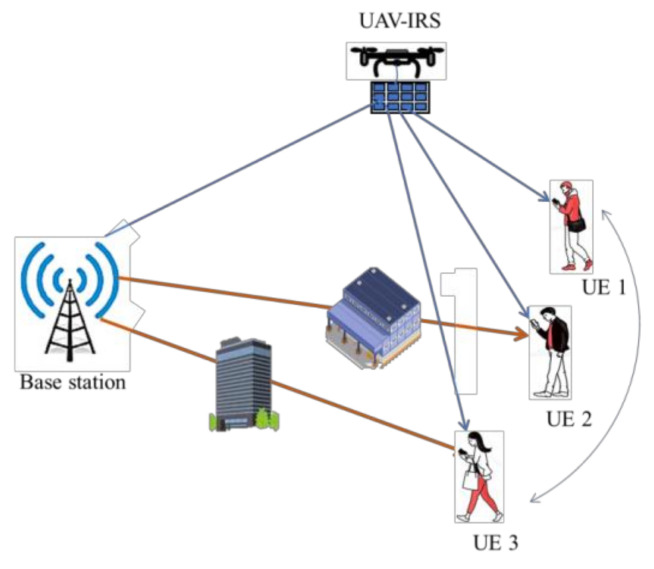
Illustration of a UAV-IRS-assisted MEC application scenario.

IRS is a transformative technology to improve the computing performance gains [84]. IRS passive beamforming puts a huge impact on offloading policies of users to the communication network edge along with high computing power to ensure better resource allocation and reduced computation latency. For example, some users located in nearby server can significantly alleviate congestion and offload traffic to a remove server through IRS reflection links in order to achieve cost-effectiveness, low power consumption, and reduce computation latency. THz communication has high stringent requirements for latency and support higher data rate, therefore, developing MEC in THz can mitigate impediments such as excessive offloading delays. However, this integration of MEC and THz suffers greatly from unstable offloading in THz, which impacts latency and power consumption. In such scenario, IRS can be utilized to overcome latency and throughput issues. In [85], the authors consider IRS-assisted MEC system to improve the channel conditions. Some recent studies have focused on green edge interference to reduce power consumption for data transmission and computation [86,87]. Even though these works highlight optimization methods for IRS-assisted MEC, these systems have not yet been fully explored for THz communication. These optimization strategies will be more complex for IRS-assisted THz MEC as it requires high computing capabilities, low system utility, and stable transmission. Stated thus, the aforementioned issues should be handled gradually. In [18], the authors address the downlink communication of a multi-antenna BS along with single-antenna user equipment (UE) supported by UAV to satisfy QoS constraints and prevent service starvation for the cell-edge UE. Particularly, this study focuses on the benefits of using both IRS and UAV to support end-to-end communication along with offering high energy efficiency. It aims to maximize the total energy efficiency by the join optimization of IRS phase shift at the UAV and the beamforming at the BS. An overview of IRS-UAV assisted MEC scenario is shown in Figure 8. In [88], the authors consider a scenario where communication between BS and UE is blocked. They deploy IRS-UAV to support UE for offloading the computing task to the multiaccess edge computing (MEC) on the BS. The proposed system offers low-latency edge computing services to mobile users. The authors minimize the response time of users by jointly optimizing computational task scheduling, UAV hovering, IRS passive beamforming, and UE’s active beamforming. Another recent study [89] proposes IRS-UAV assisted mobile edge computing and traffic offloading over the RF-enabled 6G networks, which comprised of a UAV operating as an MEC server to gather data from multiple ground users (GUs) and multiple sets of IRSs to substantially improve the wireless data and energy transmission simultaneously. To tackle the hindrance of on-board energy restrictions severely impacting UAV’s performance and sustainability, authors proposed this system to reduce flying-time of UAV while empowering all GU’s data to be gathered. The processing time reduction problem was formulated by the join optimization of Gus scheduling, resource allocation, UAV flying time and trajectory and IRS phase shift. The proposed strategy can save UAV’s flying time up to 20%. 

### 3.7. Free Space Optical (FSO) Communication

Optical wireless communication such as FSO systems are promising candidates to satisfy the demands for higher data rates in B5G or 6G technology [90]. FSO systems provide wide bandwidth spectrum to support higher data rate services, e.g., video surveillance, tactile internet, and wireless backhauling. It offers unique benefits, such as easy deployment, simple system, and low-cost components. However, FSO systems are prone to several impairments in which the most critical one is strict LOS requirement between transceivers. Moreover, FSO systems face performance degradation due to misalignment, fog, heavy snowfall and atmospheric turbulence. To mitigate these challenges, different techniques have been proposed in literature including aperture averaging, beam steering, diversity techniques and adaptive optics A few studies have reported IRS-aided optical communication systems to tackle these impairments, but these studies are in sparse [91]. IRS-UAV assisted FSO communication has a great potential to construct an energy saving and flexible communication system. In [91], the authors generalized pointing error loss through Hoyt distribution and atmospheric turbulence by Gamma–Gamma distribution for performance assessment of proposed system. The proposed model was validated through Monte Carlo simulation.

#### UAV-Based IRS-Assisted RF/FSO Communication

**Figure 9:**
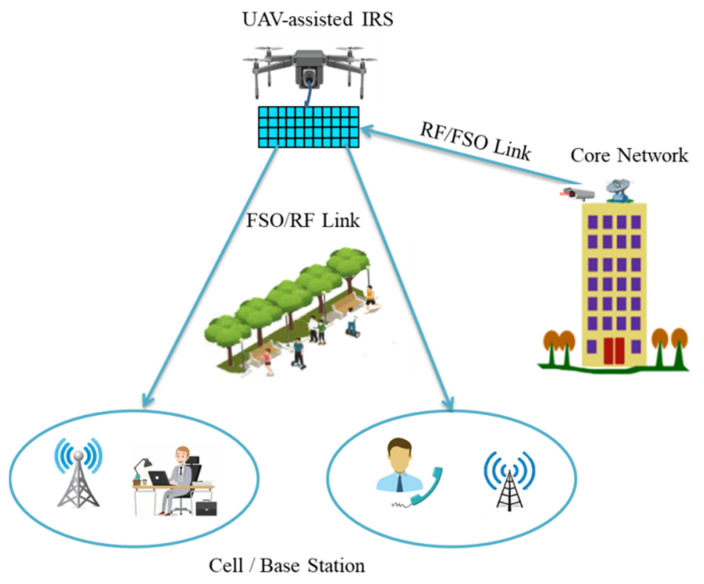
UAV-based IRS-assisted RF/FSO communication.

One of the main problems of the FSO system is the stringent LoS requirement between transceivers. A conventional method to prevent the propagation limit is to deploy optical relay nodes between transceivers. Currently, UAV-enabled relaying systems have garnered attention to mitigate LoS disadvantages. The deployment of optimal relay is a problem in FSO systems, which causes hindrance in tracking and acquisition of optical beams. A possible solution is to utilize mobile relay such as UAV which improves the reliability of optical links. Recently, a hybrid approach of RF/FSO system has gained considerable attention for IRS-assisted UAV communication. It supports both fronthaul and backhaul links for BS as they hover at high altitude and providing the LoS link. An illustration of UAV-based IRS-assisted RF/FSO system is presented in Figure 9. It combines both RF and FSO links to offer system link redundancy. In [92], the authors proposed a UAV-based IRS-assisted RF/FSO system. In the proposed system, the authors consider the phase shift error of IRS along with atmospheric turbulence. Moreover, a sophisticated model is presented for pointing error due to orientation and position fluctuations of the deployed IRS on the UAV. The authors also derived closed form expressions of spectral efficiency and average symbol rate under the combined effect of phase shift error, pointing error, and atmospheric turbulence. 

### 3.8. Internet of Things (IoT)

The key role of IoT is to support a dense connection of digital entities endowed with the potentials of sensing, actuation, and computing. A massive number of IoT devices need novels approaches, which are not fully explored in 5G wireless networks. In this respect, UAVs are envisaged to contribute towards enhancing the communication reliability and spectral efficiency of these wireless networks due to capability to improve capacity and extend coverage. Moreover, UAVs are envisioned to play a central role in information dissemination to IoT entities. UAVs take the benefits of low-cost production and mobility, being active to collect data of IoT devices. However, due to limited power and size, it is hard for UAVs to integrate advanced communication techniques to overcome the expanding demands for higher data rates. Furthermore, due to severe loss of free space, UAV missions are highly prone to power loss leading to finite capacity of UAVs. Numerous studies have addressed different aspects to overcome these challenges. For instance, some studies have reported IRS-assisted UAVs to improve data transmission between BS and IoT devices. However, these reported studies are in sparse. Particularly, these works aim to enhance data transmission and system performance considering minimal power consumption. IRS-assisted UAV communication can support energy-efficient transmission for IoT networks. It can be attained by adjusting UAV in close proximity to the energy-limited IoT devices allowing them to transmit at low power consumption in the uplink, consequently leading to prolonged battery lifetime and low energy consumption. Moreover, the use of IRS-assisted UAV communication to significantly enhance channel capacity and network coverage leads to a substantial decrease in number of BSs. In [93], the authors proposed a new IRS-assisted Master-Auxiliary-UAV powered IoT network (IRS-MAIN). IRS-MAIN does not only support wide coverage due to UAV’s mobility, but also supports improved channel condition with IRS. This study focuses on maximizing the total throughput by join optimization of the transmit power and trajectory of Master-UAV. In [79], the authors investigate the IRS-UAV-aided SWIPT for IoT networks. Particularly, an IRS carried by UAV is used to transmit information and power from the access point to multiple IoT devices. Taking full benefit of IRS-assisted UAV to serve several IoT devices flexibly, a TDMA scheduling protocol is proposed to attend multiple IoT devices alternatively during UAV flight. The presented results validate that considering a reliable and flexible IRS-assisted UAV, the minimum achievable rate of IoT network can be significantly enhanced. Similarly, the authors of [94] investigate the IRS idea in UAV empowered communications with the aim to improve communication reliability and extend network coverage, as well as spectral efficiency of IoT networks. 

## 4. Applications of IRS-Assisted UAV Communication

There are various applications of IRS-assisted UAVs in terms of communication and networking. Here, we have discussed the impact of this integration related to extended coverage, enhanced capacity, spectrum sharing, PLS, vehicular communication, smart city, cellular connected UAVs, and internet of underwater things (IoUT). 

### 4.1. IRS-Assisted UAV Communications for Extended Coverage

**Figure 10:**
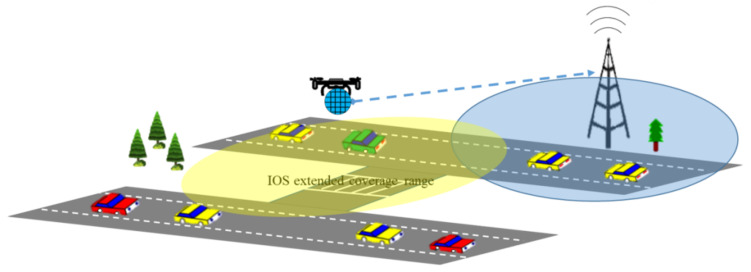
IRS-assisted UAV communication for extended coverage.

In future wireless networks, it is envisaged that aerial base station (ABS), aerial user equipment (AUE) and aerial relay (AR) will be the key enabler to achieve adaptive and dynamic coverage [23]. As active aerial communication incurs energy overhead, so IRS is a promising alternative to overcome this drawback. In such scenario, a spherical type IRS can be used to control wireless propagation at intended locations, positions, heights, and depths. It is a novel design which can reflect signal in all directions. Spherical type IRS can cover dead zones by reflecting signal in multiple directions. This 360 degree coverage can further enhance the spectral efficiency. It can support multiple features due to potential rotational mobility of IRS. Moreover, UAVs can be used to carry IRS and support aerial communication. The UAV carrying IRS can further extend coverage at suitable heights. Similarly, the network coverage can be substantially improved in any intended direction or height by optimizing IRS phase shift and UAV location and trajectory. Figure 10 presents an overview of IRS-assisted UAV for extended coverage. 

### 4.2. IRS-Assisted UAV Communications for Spectrum Sharing

**Figure 11:**
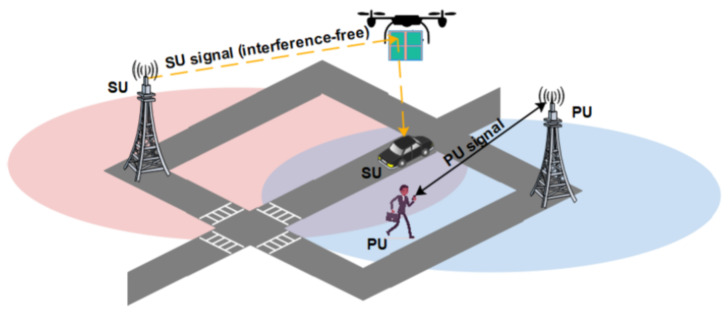
IRS-assisted UAV for spectrum sharing [23].

IRS can significantly reduce interference when devices perform transmission considering same frequency slot. It makes IRS a promising candidate for spectrum sharing feature. Mostly used conventional approaches use cognitive radios and require an effective and reliable spectrum sharing technique to mitigate interference for primary users (PUs). However, it can only be attained at the expense of reliability and energy. IRS-assisted UAVs can improve the network capacity through spectrum sharing strategy. The potentials of IRS are empirically discussed to support spectrum sharing in indoor environment [95]. In this study, IRS phase shift is used to control user interference while multiple access in spectrum sharing technique is used to increase capacity. In [95], the authors introduce IRS-aided spectrum sharing technique to improve secondary users (SU) capacity while ensuring QoS for primary users (PU) through precisely optimizing IRS phase shift to enable channel diagonalization. These works can be further modified by smartly considering UAV’s potentials to optimize the performance of wireless networks in practical applications as shown in Figure 11. Furthermore, in IRS-assisted UAV, it is essential to take account of impacts of specific parameters including altitude, latitude, and longitude of IRS phase shift to enhance network capacity. 

### 4.3. IRS-Assisted UAV-Ground Wireless Networks

**Figure 12:**
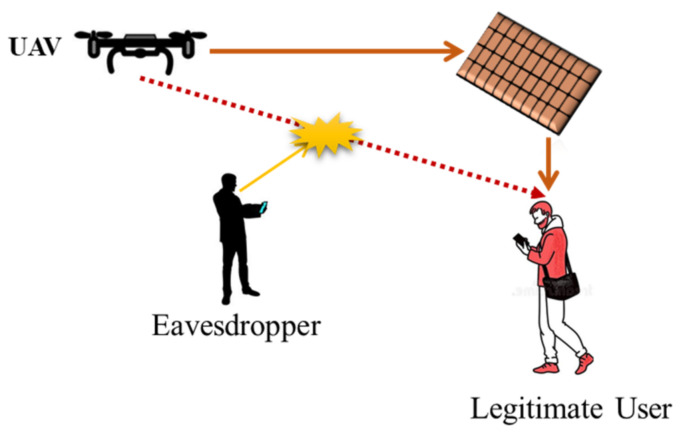
An IRS-assisted UAV system in the presence of eavesdropper.

Although UAVs can establish LoS links for communication in multiple applications, they are critically prone to blockages. In addition, UAV-based ground communication is severely degraded by interference and vulnerable to eavesdropping. To mitigate these challenges, IRS can be involved to overcome obstacles by proving reflected links and supporting the communication for the intended users. Similarly, IRS has the potential capability to deteriorate the unintended signals at the users in order to prevent data leakage and avoid interference. In general, there lies a tradeoff to jointly configure the IRS phase shift to achieve these potentials. In a recent study [24], authors report IRS-assisted UAV communication to enhance the quality of intended signals and suppress the unintended signals respectively. An overview of IRS-assisted UAV is presented in Figure 12 in the presence of an eavesdropper to validate the potentials of IRS by improving the security of UAV-based ground networks. 

### 4.4. IRS Enhanced PLS for UAV Communications

**Figure 13:**
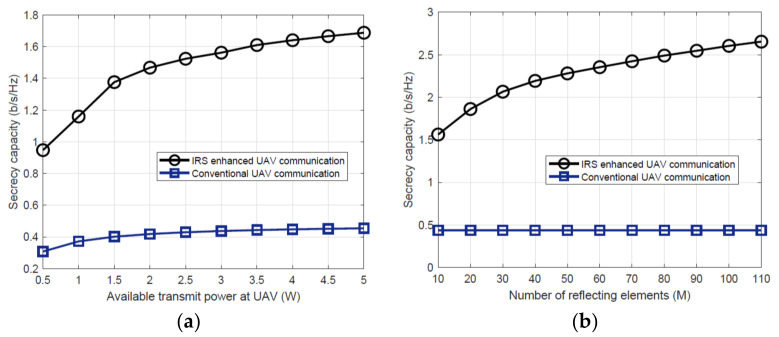
Secrecy capacity of IRS-assisted UAV system (**a**) impact of increasing transmit power at the UAV and (**b**) impact of increasing number of increasing elements of IRS [96].

UAVs show the capabilities to enhance the physical layer security (PLS) for terrestrial wireless communication. It is conceived by establishing a dominant LoS link between ground and aerial nods. PLS can significantly support the received information at the legitimate users than eavesdroppers. Effective PLS can be achieved through various methods such as power allocation, jamming, coding, and signal processing techniques [96]. PLS can effectively support defense, authentication, and key generation against eavesdropping. Some potential characteristics of PLS for security are low computation cost and packet size. Thus, PLS appear to be suitable candidate for energy constraint devices like UAVs and IoT terminals. UAVs can serve as ARs between legitimate nodes to weaken the communication link for eavesdroppers. Similarly, UAVs can send artificial noise towards eavesdroppers as friendly jammers to prevent legitimate users from any possible attack. IRS can also support data confidentiality of UAV communication through enhance secrecy rate. IRS passive reflecting elements can cause destructive reflection to restrict the eavesdropper and reduce the received SNR. Apart from security, IRS-assisted UAV can support reliable and energy efficient communication, which ultimately enhances the battery endurance of UAVs. In a recent study [96], the authors validated the impacts on secrecy capacity by increasing both transmit power at the UAV and the number of reflecting elements at the IRS. Figure 13a shows that by increasing the available transmission power at the UAV, the secrecy capacity also increases. It can be noticed that a significant gain can be achieved at lower values of transmit power. Similarly, Figure 13b shows the impact of increasing the number of reflecting elements of IRS which results in enhanced secrecy capacity. We have summarized some recent studies on IRS enhanced PLS for UAV communications in Table 6. 

### 4.5. IRS-UAV Enabled Smart City

**Figure 14:**
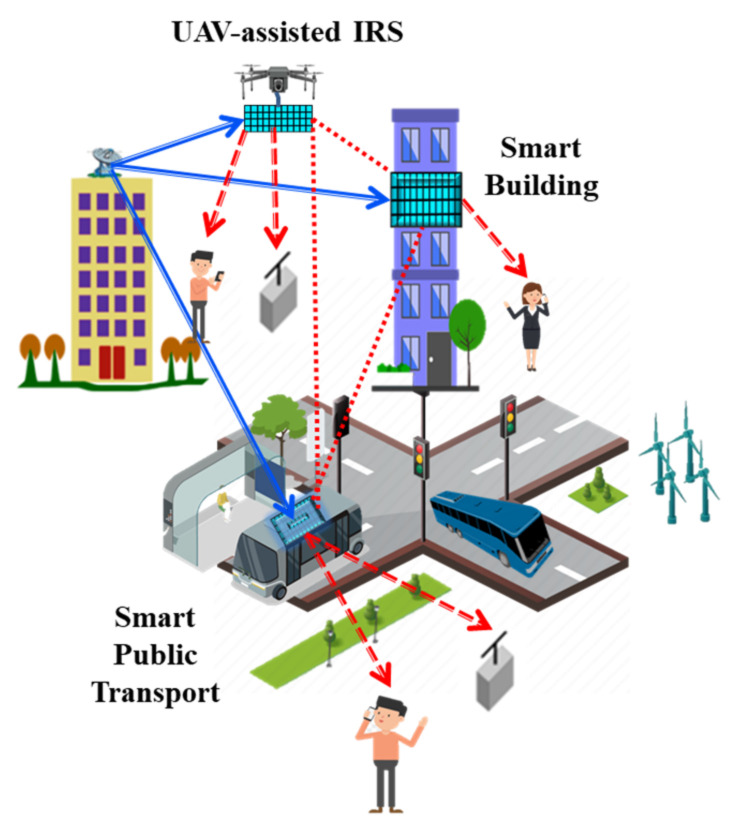
An overview of IRS-empowered UAV in smart cities (modified from [76]).

The idea of smart city has emerged from the harmonization of digital technologies and digital transformation of several ecosystems. In future, wireless networks must be smartly designed for optimal use of city resources. The concept of smart city can support accessibility to public services, advance digitalization of urban scenarios and advance monitoring capabilities of societal processes. In the foreseeable future, smart city elements will be more configurable and unified with the capability of self-management. Recently, “smart radio environment” was proposed in a study [101] for better performance of wireless communication. It is closely related to the current ongoing developments of IRSs. Therefore, IRSs are envisioned to support required flexibility in “smart radio environment” or “smart communication environment” through energy and cost-efficient wireless propagation. Through employing IRSs, smart city ecosystems will be more configurable and adaptable, which will subsequently support easy installation of future wireless networks in urban environments and can further boost the interconnectivity among public and private applications. S. Kisseleff et al. [102] discussed the innovative concept of IRS-assisted smart cities by considering several use cases, applications and key benefits. According to the authors, the basic architectural elements of a smart city will be autonomous vehicles, communication platforms, sensors/actuators, and services/applications. They also identified relevant applications as smart homes, smart buildings, smart factories, smart hospitals, smart billboards, and smart communications. In this study, the authors also investigate several research opportunities and future directions. They addressed key-enabling aspects if IRS-UAV assisted smart city requiring substantial research contributions in pilot decontamination, precoding for large multiuser networks, distributed operation and IRS phase control. It is envisaged that IRS-UAV assisted smart city will support enhanced security, better resource allocation and high QoS. An overview of IRS-UAV assisted smart city is presented in Figure 14 where IRS can be installed at various sports such as mobile vehicles, UAVs, or fixed buildings. Some key benefits of using IRS-UAV in smart city are reduced energy consumption, smart communication, and high coverage. It can also tackle health, environmental, and ecological concerns with the proliferation of wireless technologies, specifically operating in high spectrum mmWave and THz bands and relieving human exposure to harmful RF radiations. 

### 4.6. IRS-Assisted UAV Vehicular Communication

Vehicular networks are extensively investigated to feature cutting-edge applications like intelligent transportation systems (ITS) and autonomous or self-driving vehicles. The vehicular networks are expected to offer reliable communication, low latency, and high data rate for ITS. Multiple applications of vehicular communication have been studied for autonomous, control, safety, and comfort-oriented features. For example, laser imaging detection and ranging (LIDAR) technology is being used in vehicular communication for securing Gb/s data rates. Researchers have also reported 10–300 GHZ mmWave for vehicular communications [103]. However, mmWave communication is highly vulnerable to obstacles and high path loss. Moreover, transmission outage and beam tracking are not yet fully explored in mmWave communication. Furthermore, extreme narrow beams of mmWave lead to critical problems of secrecy information leakage and legitimate user blockage due to misalignment. THz communication can be another future technology for terabit vehicular networks in the domain of wireless networked smart automobiles. The THz beam’s alignment and stability can be hampered by the dense mobility of people and fluctuating congested traffic. To mitigate these challenges and to ensure reliable communication, IRS has been used as a cost-effective solution to extend coverage, reduce blockage and strengthen signal quality by passive reflecting elements. In order to support THz beam training and tracking in traffic congested areas, the mobile IRS carried by UAVs can help in traffic flow. The IRS carried by UAV can be positioned at different places, positions, and heights. In order to ensure a steady, real-time and high-speed THz communication, vehicles can pick cooperative IRS-UAV on blockage conditions at different places. It is envisaged that IRS- assisted UAV communication can support traffic monitoring, traffic accident prediction and avoid traffic congestion. In [104], AU Makarfi et al. proposed IRS-assisted vehicular network to enhance PLS. The authors evaluated the performance of proposed architecture in terms of secrecy outage probability (SOP) and average secrecy capacity (ASC). The authors also studied the impact of several parameters and presented analytical expressions for the two performance metrics. In [105], J. Wang et al. briefly discussed outage analysis for IRS-aided vehicular communication. The authors derive the numerical expression for outage probability by using central limit theorem and series expansion. The presented results indicate that IRS can effectively reduce the outage probability for vehicles located in its vicinity. Better performance can be further achieved by using a higher number of reflecting elements. 

### 4.7. IRS-Assisted UAV Communication in IoUT

**Figure 15:**
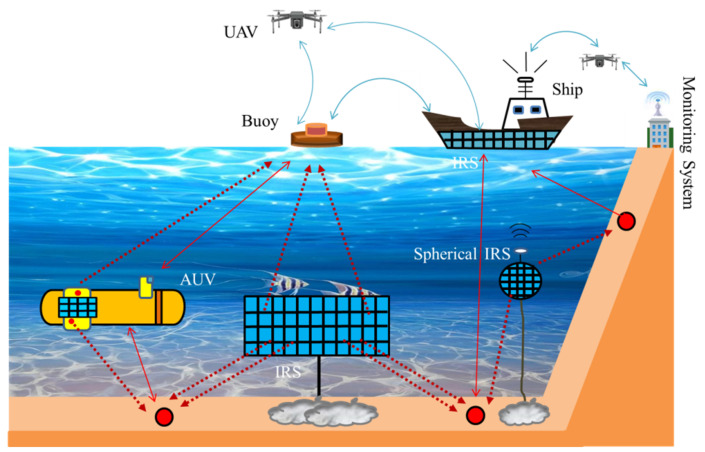
IoUT assisted by IRS-assisted UAV, motivated by [107].

In underwater medium, optical and electromagnetic (EM) waves restricted to a short range while acoustic waves are used to achieve larger range in the context of Internet of Underwater Things (IoUT). On the other hand, acoustic communication is critically vulnerable to water species, water streams, suspended particles, and scattering at uneven surfaces. It also suffers from high path loss and significantly reduces the effective data rates. To tackle these shortcomings, IRS has been proposed as a viable solution. An application scenario of IRS-assisted UAV communication is presented in Figure 15 for underwater applications. IRS can significantly reduce the multipath effect through beam steering. By optimizing IRS reflection elements, frequency selectivity can be reduced which leads to improved signal bandwidth. Below the water surface, IRS can be places in different geometries and locations. IRS can be located on the seashore, and can be installed on a ship or moving AUVs. AUVs can be preferred to carry IRS to preserve control on the smart environment and reduce any vulnerability. This smart integration of IRSs and AUVs can support better performance through dynamic mobility of AUVs and beam steering through IRS phase shift [106]. Furthermore, UAVs can fly above the water surface to gather data from ships or buoys and transmit to remote monitoring systems. IRS-assisted UAV communication requires further research efforts to fully realize this strategy to support smart ocean transportation, harbor monitoring, disaster prediction, data collection, and underwater communication. 

### 4.8. Cellular Connected UAVs

UAVs can be regarded as mobile relaying systems or virtual base stations which can be installed in various applications in multiple ways to support large scale coverage of B5G and 6G wireless networks. B5G and 6G networks are planned to deploy the terahertz communication band in order to achieve enhanced traffic between UAVs and users as well as BSs to fully harness the capabilities of UAVs. UAV cause severe interference from/to a massive number of co-channel BSs. However, due to the observable characteristics of air medium, THz communications, which rely on high power beamforming to combat challenges like fading, are highly vulnerable to mutual interference, consequently reducing transmission performance. In such conditions, IRS can be installed to smartly control the beam’s direction from the base station to ensure a variety of UAV connections. It provides as easy technique to control the reflection angel of incident signals to achieve propagation in a desired direction. Primarily IRS is integrated on roof or outer walls of buildings in urban environments. It can significantly enhance the interconnectivity of air-to-ground communication while preventing from multi-UAVs interference by introducing it into the BS beamforming scenario. In a recent study [108], authors introduced an efficient scheme to mitigate interference by deploying IRSs near the BSs. Particularly, in the uplink, IRS passive beamforming support to enhance/suppress the UAV interference to facilitate the linear or non-linear interference mitigation at each BS. While in the downlink, the IRS passive beamforming can suppress interference of BS to the UAV. The presented results outperform benchmark schemes and provide significant design insights for IRS-assisted UAV communication in cellular networks. 

### 4.9. UAV Placement/Trajectory Optimization

For terrestrial IRS, the UAV trajectory/position should be jointly optimized with the IRS passive beamforming, which is a new critical issue to be addressed. 

Suppose first the 3D UAV deployment design for quasistatic UAVs. For urban areas with high rise dense buildings, the UAV is required to be located at a sufficient high altitude in order to establish LoS links with the ground users. However, increasing the altitude for UAV leads to high path loss, which brings a basic tradeoff between LoS probability and path loss. Interestingly, IRS can be deployed to alleviate this tradeoff. For instance, by integrating an IRS near the cell-edge users having low LoS probability with the UAV, the UAV altitude can be significantly reduced. This, therefore, supports reducing the link distance between the users and UAV. As a result, it also reduced the path loss, while maintaining the rate performance of IRS-aided users. Moreover, for high mobility UAVs, the UAVs trajectory should be jointly optimized with the IRS passive beamforming to gain the optimal performance. Usually, IRS performs well when it is located near the transmitter or receiver by minimizing the product distance path loss. It offers new insights for UAV trajectory to be optimized for IRS-assisted communication. For instance, when IRS is installed near ground-based users, there are other ground users which are not located in IRS’s coverage range. To enhance the minimum data collection rate, the UAV without IRS is required to fly towards every user to enhance the communication channel. On the other hand, with deployed IRS, the UAV is not required to fly towards IRS-assisted users and, thus, can spend more time to serve other network users. Alternatively, IRSs can also be integrated near the UAV location where UAVs are installed; thus, LoS links with the target ground users can be established. In such case, the UAV can visit the nearby IRSs without visiting the far-away users, hence reducing the flight-time and propulsion energy consumption for UAVs [25]. 

### 4.10. IRS-Assisted UAV Communication in Underground Scenario

To satisfy the requirements of exceptional wireless communication service at anytime and anywhere, the high-speed and high-reliability data services in tunnel, e.g., mine tunnels, road tunnels, and subway tunnels, are required [109]. However, restricted by Snell’s law, the blockages (such as rockfall or mining equipment due to geological hazards) in underground scenario or tunnels can cause ray-link blocking. The mitigation of these blockages in tunnels is highly important to ensure reliable communication for search and rescue tasks in case of tunnel disaster. To overcome such challenges, IRS is proposed to mitigate the challenge of signal blocking in blocked tunnels or mines [110]. 

One of the internet-of-underground-things’ (IoUgT’s) primary application scenarios is the installation of sensor nodes in mines and tunnels. The uncontrolled reflections from the tunnel or mine walls and dispersion at the borders of the walls provide the biggest obstacle to the propagation of EM signals. This causes the degraded performance of network’s connection and signal quality. This problem may be solved by installing IRSs in the tunnel’s walls and ceiling, which will increase the directivity of signal transmission by guiding the signal in the direction of the passageway. Additionally, as was previously indicated, the earth may somewhat absorb the signals, meaning that each reflection may add to the overall route loss. However, in order to prevent further losses that would otherwise make the usage of IRS inefficient, IRSs are normally constructed with the maximum reflection coefficient. Due to the fact that the IoUgT is not moving, the passage map can be used to estimate the ideal position and rough values of the phase shifts. Moreover, UAVs can also be used to support in such underground scenarios such as establishing communication link, video shooting, and capturing images in search and rescue missions. 

### 4.11. IRS-Assisted UAV Communication in Smart Industry

The Industrial Internet of Things (IIoT) bridges the physical and digital worlds to improve productivity and efficiency in smart manufacturing [111]. The IIoT links millions of industrial entities to the internet in order to ensure efficient productivity and reliability. This architecture is a component of the broader technological advancement known as Industry 4.0. The integration of smart technologies will turn conventional industrial control and automation techniques into cyber-physical manufacturing units. 

Industrial wireless communication systems are highly vulnerable to thick building structures, room dimensions, arbitrary mobility of objects, EM interference, and metallic structures. In contrast, full industrial automation needs low latency and ultra-reliability to support efficient mission-critical industrial applications. Furthermore, human–machine interaction in Industry 5.0 will lead to more complexity. Similarly, the growing demand of several emerging services in innovative industrial sectors such as holographic control display and augmented reality (AR) or virtual reality (VR) maintenance will create more challenges in future industries. To overcome the communication challenges of such emerging services, Industry 5.0 needs to integrate promising technologies including efficient energy harvesting, advanced localization, terahertz, and mmWave communications [111]. 

To overcome aforementioned impediments and meet the requirements of future smart manufacturing industries, IRS can be a viable solution. IRS can be used to steer the signals towards target nodes and avoiding obstacles. IRS can be used to mitigate the interference caused by several production processes. Moreover, some manufacturing processes and machines are mobile and several machines are required to make same copies of specific products. In such cases, IRS-assisted UAV network can be used to support these manufacturing services. IRS-assisted UAV system will have a broader view of manufacturing process.

## 5. Open Challenges and Future Research Directions

In this section, we elaborate some research challenges and opportunities which are essential to leverage the capabilities of IRS-assisted UAVs for both design and implementation. 

### 5.1. Physics and EM Compliant Models 

In literature, IRSs are mostly considered as good reflectors with ideal phase shifts and perfect manipulator of incident EM waves. However, these surfaces should consider other parameters as well rather than only reflection. It is a paramount significance to consider the reliability and functional capability with varying communication frequencies. The ME response of IRS is associated to factors including polarization, incident and reflection angle, hardware design, and designing material. The properties of IRS also depend upon its size and diverse number of reflecting elements. Thus, apart from basic models with realistic performance, EM compliant and physics modeling [112] should be taken into account. Usually, proper analysis of shadowing, scattering, and fading effects are required for channel modeling. For accurate channel modeling for IRS-assisted UAV, IRS geometry, fabrication, orientation, and number of reflecting elements should be kept into consideration. Both IRS and UAV define channel models as sophisticated or challenging [23]. For instance, a UAV with dynamic mobility and shadowing occurred due to this mobility causes wide spatial or temporal alterations. This dynamic mobility of UAVs causes beam misalignment and fluctuations which cannot be in neglected in high frequency communications. Other factors should also be analyzed, such as atmospheric attenuation and reflection loss. Furthermore, IRS also impacts channel modeling because of its scattering elements and near-field propagation. 

### 5.2. Channel Sensing and Estimation

**Figure 16:**
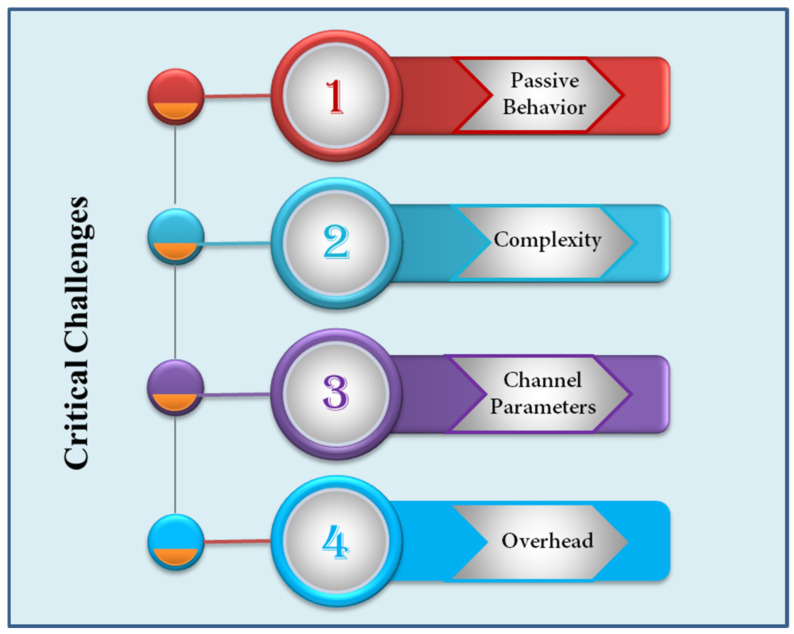
Crucial challenges in IRS channel estimation.

IRS contains massive numbers of reflecting elements which are attached with a centralized controller. The application of IRS is based on these reflection elements to attain optimal beamforming through controlled radio propagation. However, it can be achieved through channel sensing and signal processing. Without proper signal processing, it is difficult to obtain channel sensing. On the other hand, channel estimation delay and accuracy should be kept into account. Existing studies focus on sophisticated signal processing techniques for low overhead and enhanced accuracy. However, these techniques usually require high power consumption to execute task, data processing and transmission. Thus, it is critically essential to investigate sustainable channel estimation techniques. One possible solution is to use IRS with low-power sensing devices which can perform channel sensing and estimation. Other option is to utilize ML tools. The availability of accurate channel state information (CSI) acquisition is very important to perform perfect reflection for IRS-assisted UAV communication [24]. The performance of beamforming gain degrades in case of imperfect CSI. To gain such information within channel’s coherence time in mobile environment is a critical challenge. ML and DL approaches can be utilized to solve CSI problem by channel estimation. However, these technologies usually require longer training time and vast amount of data. Thus, delay impact due to CSI estimation and distance should be investigated. Fast and precise CSI acquisition is mostly preferred. Hence, research efforts are needed to investigate accurate channel estimation without high overhead and high power consumption in channel training. Figure 16 outlines some critical challenges for channel estimation of IRS-assisted UAVs. 

### 5.3. Controller and Overhead of IRS

It is crucial to properly control the reflecting elements of IRS to obtain required phase shift through a controller. In general, a robust, fully synchronized and highly reliable controller is selected [23]. Researchers have also proposed Control link can be easily established in static use case, but it faces fading, shadowing, and time-dependent channel characteristics for IRS-assisted UAV communication. Researchers have been focusing on investigating novel strategies to control signaling, processing overheads to achieve reliable and stable link performance. One possible solution is proposed to utilize UAV smarms with communication capabilities and distributed computing to support availability and reliability of control links. Another solution is to use ML controllers to optimally adjust the IRS phase shift. However, these tools can impose additional constraints on the UAV’s resources in the aspect of power and memory consumption. 

### 5.4. Power Consumption 

An IRS mostly requires an energy supply due to the absence of power amplifier [24]. On the other hand, energy conservation of UAV is also critical due to deficient battery endurance. It tends to be a critical bottleneck on UAV performance, flight time and battery life. One solution is to wirelessly charge UAVs while flying. WPT techniques can be adopted, where another UAV can be used to transfer required energy for mission continuity. Thus, researchers should develop energy-efficient mechanisms and suitable optimization frameworks to reduce power utility without comprising over the IRS-assisted UAV communication performance.

### 5.5. Environmental Factors 

In existing works, researchers consider stable flight of UAV and users located at fixed positions. However, it is not realistic in practical scenario. In real-world use cases, UAVs suffer from inventible jittering caused by wind and vibrations, tending to unstable performance and trivial channel estimation errors. As a result, it becomes difficult to completely exploit the joint beamforming design gain. In addition, the airflow can alter the UAV’s speed and trajectory, leading to performance detriment and safety concerns. It is also hard to meet QoS requirements under dynamic environment and varying locations. 

### 5.6. Design of Suitable Security Measures

UAVs are highly vulnerable to several types of attacks, both from physical and network point of view. Researchers can exploit the benefits empowered by IRS-assisted UAVs to improve the security of deployed UAVs. In IRS-assisted UAVs, mobility and high flexibility are important factors to restrict potential eavesdroppers, protect data leakage of legitimate users and improve PLS [96]. Particularly, it is critically important to secure the sensitive data of legitimate UAV which can be intercepted by a malicious UAV. This research domain is at infancy and advanced security mechanism should be investigated to improve security against eavesdroppers. 

In contrast to conventional UAV communication, IRS-assisted UAV communication moves one step ahead to improve PLS after optimizing IRS beamforming and phase shifts of passive reflecting components. Since IRS, UAV, and associated resource optimization (i.e., power allocation, sub-carrier, trajectory design, etc.) are customarily linked, the design optimization challenge appears to be inflexible, and the current designs appear to be sub-optimal [96]. Nonetheless, the performance variations between these existing sub-optimal and optimal designs are not fully evident. Thus, research fraternity is focusing to find optimal approaches to improve PLS of IRS-assisted UAV system in various scenarios while maintaining system performance and computational intricacy. 

### 5.7. Machine Learning/Artificial Intelligence Techniques 

The optimization of IRS-assisted UAV communication is important when UAVs are deployed in unpredictable and hard environments such as heavy wind or rain. In particular, optimization of UAV trajectory, IRS phase shift and resource allocation is difficult due to non-linear models. Thus, it is suggested to find new design strategies with low intricacy and efficient performance. In this regard, AI and ML tools are promising approaches to efficiently design and optimize these networks. These approaches are based on reliable, secure, and robust tools to optimize challenging environments. Furthermore, hybrid online and offline techniques, hybrid models, and data-driven models can be utilized to analyze intricate networks for secrecy performance enhancement. However, different parameters are still needed to be explored—for instance, high energy consumption, latency, throughput and computational processing power.

### 5.8. Medium Access Control Layer

The integration of IRS-UAV in any multiple users’ environment will have a great significance to improve the performance of future wireless networks. Particularly, developing artificial intelligence based medium access control methods for mmWave and THz communication considering the impact of physical layer security is a critical issue which must be addressed. Additionally, the deployment strategy of IRS in multiple users’ environment requires innovative AI-empowered strategies such as transfer learning frameworks and multi-agent RL to jointly optimize PHY and MAC layer.

## 6. Conclusions 

This study offers a comprehensive review on the existing literature on IRS-assisted UAV communication and also discusses some new ideas. We discussed both ground and airborne scenarios for IRS-assisted UAV communications. In this study, we discuss several applications which can be achieved by IRS-assisted UAV communications. It is envisioned that due to high reconfigurability, high mobility, low energy consumption, and low expenditure, the IRS-assisted UAV communication will expand both terrestrial and airborne wireless networks. It can ensure secure transmission and improve wireless network throughput. We briefly discuss the integration of emerging technologies, such as machine learning, deep learning, mmWave, THz, FSO, VLC, MEC, NOMA, and WPT, for breakthrough novelties of IRS-assisted UAV communication. We present critical challenges arising from the integration of UAV and IRS with solutions and directions how to mitigate them through focused research. This study also highlights open challenges and future research directions. We believe this article will pave a way for further research contributions by providing guidance for theory, research, experimental demonstrations, and real-time implementation. 

## Figures and Tables

**Table 1:** Existing works combining UAV and IRS.

Reference	Aim	Optimization Variable	UAV Mobility	IRS Installation
[12]	To maximize the average achievable rate	UAV trajectory, IRS passive beamforming,	Mobile	At the building
[15]	To maximize the IRS data transmission	IRS scheduling, IRS phase shift, UAV trajectory	Mobile	At the building
[19]	To maximize the received power	IRS passive beamforming, beamforming and trajectory of UAV	Mobile	At the building
[20]	To maximize the secrecy rate	IRS phase shift, power control of UAV, trajectory	Mobile	At the building
[21]	To maximize the rate of strong user	Location, phase shift and beamforming of IRS-UAV	Static	At the UAV
[22]	To maximize the transmission capacity	Reflection and location parameters of IRS-UAV	Static	At the UAV

**Table 2:** IRS-assisted UAV communication scenarios.

Reference	System Components	Channel Model	Design Characteristics	Metric
[13]	UAV, IRS, user	Line-of-sight	UAV trajectory and velocity, IRS phase shift	Average total power consumption
[15]	UAV, IRS, BS	Rician	UAV trajectory, IRS scheduling, IRS phase shift matrix	weighted sum BER minimization
[19]	UAV, IRS, user	Rician, Raleigh, LOS	UAV trajectory, IRS phase shift, linear precoding	Signal-to-noise-ratio (SNR)
[20]	UAV, IRS, user	Rayleigh, free-space pathloss	UAV trajectory, IRS phase shift, linear precoding	Average capacity, average BER, Outage probability
[29]	UAV, IRS, mobile users	3GPP	UAV trajectory, IRS phase shift, precoding	Energy consumption minimization
[31]	UAV, IRS, user	Rician	UAV trajectory, IRS phase shift	Average energy consumption
[33]	UAV, IRS, user	Multipath channel	UAV trajectory, IRS phase shift, precoding, analogue beamforming, user scheduling	Sum rate
[34]	UAV, IRS, user	mmWave channel	UAV trajectory, IRS phase shift, precoding	Sum secrecy rate
[35]	UAV, IRS, user	Rician, LOS	UAV trajectory, IRS phase shift	Bit-error-rate (BER)
[36]	UAV, IRS, user	mmWave channel	UAV trajectory, IRS phase shift	Weighted data rate and geographical fairness

**Table 3:** Different techniques to realize reconfigurable metasurfaces.

Reference	Tuning Technique	Material	Characteristics	Spectrum	Modulation Range/Speed
[39]	Capacitance	PIN diode/varactor	Reprogrammable Hologram/Tunable lens	MHz-GHz	NA/100 KHz
[40]	Mechanics	NEMS/MEMS	Modulator	GHz-visible	31%/1 KHz
[41]	Phase transition	VO_2_	Modulator	THz-visible	20%/NA
[42]	Phase transition	Liquid crystals	Color filter/beam deflector/modulator	GHz-visible	12°/NA
[43]	Carrier doping	Graphene	Absorber/polarizer	THz-NIR	243°/NA
[44]	Carrier doping	Semiconductors	Modulator	THz-visible	90%/NA

**Table 4:** Academic and industrial projects on intelligent reflecting surfaces.

Year & Reference	Research Project	Aim/Objective	Product
2017 [48]	VisorSurf	It aims to develop a hardware architecture for software-driven metasurface	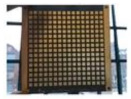
2017 [49]	Reconfigureable active Hygen’s metalens	To achieve efficient manipulation of the impinging wavefront	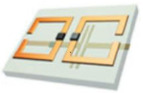
2018 [50]	NTT DOCOMO and Metawave	To support 5G data transmission of 28 GHz-band using metasurfaces reflectarray	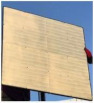
2020 [51]	Rfocus	Largest number of antennas used for for a single link communication	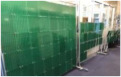
2020 [52]	RIS-based MIMO QAM	To demonstrate an RIS framework to attain amplitude-and-phase-varying modulation, which supports the architecture of MIMO quadrature amplitude modulation (QAM) transmission.	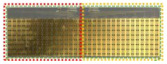
2020 [53]	NTT DOCOMO and AGC Inc.	It aims to design first ever prototype of transparent dynamic metasurface for 5G.	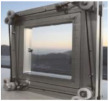

**Table 5:** Research contributions on UAVs.

Reference	Year	Research Focus
[57]	2020	This study focuses on UAV applications, challenges, regulations, and future research aspects. Specifically, it highlights issues regarding trajectory, energy harvesting, security, interference and collision avoidance.
[58]	2020	This study is focused on UAVs for three different perspectives including swarms, sensors and communications.
[59]	2020	This study focuses on several application scenarios of multi-UAV systems. Additionally, it highlights nomenclature taxonomy, architecture, current trends and potential challenges.
[60]	2021	This article comprehensively surveys green UAV communications, energy consumption models, applications, common trends and research challenges.
[61]	2021	This article focuses on UAV prototype, experimental demonstration, channel models and energy consumption models. Moreover, it also outlines various future research directions for UAVs.
[62]	2021	This work is based on deep learning tools to detect vehicles using UAV aerial images. It addresses optimization methods, reduction of computation overhead and accuracy enhancement. This work provides guidelines for researchers in artificial intelligence and traffic surveillance domains.
[63]	2022	This study focuses on optimization algorithms e.g., Chicken Swarm Optimization Clustering, bee optimization algorithm, and genetic algorithm which are the gateway to better reliability, performance and accuracy. It also addresses protocols, routing schemes and associated challenges.
[64]	2022	This study surveys various task assignment algorithms in the context of main ideas, benefits, drawbacks and operational features. These algorithms are compared on the basis of performance factors and characteristics. This study also discusses challenges, open issues, and possible future research directions.

**Table 6:** Existing works on IRS enabled PLS for UAV communication [96].

PLS Aspect	UAV Aspect	IRS Aspect	Scenario	Results
Maximize secrecy rate [20]	Power control, trajectory control	Phase shift control	Single eavesdropper, UAV to single receiver	Enhanced secrecy rate
Maximize secrecy rate [76]	Position design, Beamforming design	Position design, beamforming design	Single eavesdropper, UAV BS to receiver	Enhanced secrecy rate
Maximize secrecy rate [97]	Position design, power control	Phase shift control	Single eavesdropper, UAV to ground user	Enhanced secrecy rate
Maximize secure EE [98]	Trajectory design, power control	User association, phase shift control	Single eavesdropper, IRS equipped UAV, BS to users	Enhanced secure EE
Maximize secrecy rate [99]	Trajectory design	Beamforming design	Single eavesdropper, UAV to ground user	Enhanced secrecy rate
Maximize secrecy rate [100]	Position design	Phase shift control	Single eavesdropper, BS to users	Enhanced secrecy rate

## Data Availability

Not applicable.
